# Testing the short‐term effectiveness of primary care referral to online weight loss programmes: A randomised controlled trial

**DOI:** 10.1111/cob.12482

**Published:** 2021-10-06

**Authors:** Michaela Noreik, Claire D. Madigan, Nerys M. Astbury, Rhiannon M. Edwards, Ushma Galal, Jill Mollison, Fitsum Ghebretinsea, Susan A. Jebb

**Affiliations:** ^1^ Nuffield Department of Primary Care Health Sciences University of Oxford, Radcliffe Observatory Quarter Oxford UK; ^2^ NIHR Oxford Biomedical Research Centre Oxford University Hospitals, NHS Foundation Trust Oxford UK; ^3^ Centre for Lifestyle Medicine and Behaviour Loughborough University Leicestershire UK

**Keywords:** digital intervention, digital weight loss programmes, obesity

## Abstract

Guidelines ask health professionals to offer brief advice to encourage weight loss for people living with obesity. We tested whether referral to one of three online programmes could lead to successful weight loss. A total of 528 participants aged ≥18 years with a body mass index of ≥30 kg/m^2^ were invited via a letter from their GP. Participants were randomised to one of three online weight loss programmes (NHS Weight Loss Plan, Rosemary Online or Slimming World Online) or to a control group receiving no intervention. Participants self‐reported weight at baseline and 8 weeks. The primary outcome was weight change in each of the active intervention groups compared with control. We also compared the proportion of participants losing ≥5% or ≥10% of body weight. For Rosemary, Online mean weight loss was modestly greater than control (−1.5 kg [95% confidence interval (CI) −2.3 to −0.6]) and more than three times as many participants in this group lost ≥5% (relative risk [RR] = 3.64, 95% CI: 1.63–8.1). For Slimming World, mean weight loss was not significantly different from control (−0.8 kg [95%CI −1.7 to 0.1]), twice as many participants lost ≥5% (RR = 2.70, 1.17–6.23). There was no significant difference in weight loss for participants using the NHS Weight Loss Plan (−0.4 kg, [95% CI −1.3 to 0.5]), or the proportion losing ≥5% (RR = 2.09, 0.87–5.01). Only one of three online weight loss programmes was superior to no intervention and the effect size modest among participants living with obesity.

**What is already known about this subject?**

- Until recently face to face group‐based programmes have been the mainstay of weight management services, but there has been a rapid rise in the availability of digital interventions, further accelerated by the COVID‐19 pandemic.
- Systematic review evidence shows that digital interventions result in effective weight loss but there is considerable heterogeneity which may arise either because of differences in intervention components or participant characteristics.
- Little is known about the effectiveness of referrals to digital interventions with little or no in‐person contact.

**What this study adds?**

- This study shows that referral to the tested online weight loss programmes is, at best, only marginally superior to no intervention.
- Low or no weight loss in the intervention groups emphasises the need to carefully monitor the outcomes of programmes to encourage continual improvement.
- Low uptake to the intervention should prompt caution in using letters to recruit participants to weight loss interventions in routine care.

## 1 INTRODUCTION

In the UK, more than a quarter of all adults have a BMI ≥30 kg/m^2^ and three‐quarters of these people are seeking to lose weight.^1^ Health professionals are encouraged to offer advice to lose weight to people living with obesity, but while self‐directed weight loss can be effective, the magnitude of weight loss achieved is typically less than occurs when following a structured programme.^2^ Until recently face to face group‐based programmes have been the mainstay of weight management services, but there has been a rapid rise in the availability of digital interventions, further accelerated by the COVID‐19 pandemic. These have the potential to be more easily scalable and could be offered at a low unit cost.

Digital weight loss programmes include those designed to be used with an “app” for a mobile phone or tablet and those that are web‐based. These programmes usually aim to encourage a healthier, low‐energy diet, and to increase physical activity. The behavioural strategies vary widely, but commonly include self‐monitoring of behaviours/outcomes, goal setting, automated reminders or feedback, or social support via text, or social media. A notable distinction between programmes is the level of personal contact and individualised coaching on offer. A review of technology‐based interventions concluded that incorporating individualised feedback from an interventionist is an important component of successful technology‐based weight loss interventions.^3^ However, including remote support to digital programmes, adds to the cost and complexity of the intervention, reducing some of the potential advantages of digital programmes.

The objective of this study is to determine the effectiveness of largely unsupported digital interventions for weight loss in a primary care setting. We selected three popular and easily accessible digital weight loss programmes; one provided free of charge by the NHS in England and two from commercial providers, which were available in an online format.

## 2 MATERIALS AND METHODS

### 2.1 Study design

This study was an individually randomised four‐arm, parallel design, open‐label, controlled superiority trial. Eligible participants were adults aged 18 years or above, with a body mass index of ≥30 kg/m^2^, who had access to a mobile phone with web capabilities, the internet, and weighing scales. Participants were excluded if they were already following a weight loss programme (defined as a structured, prescribed and monitored programme, and not a self‐regulated diet), were pregnant, breastfeeding, or planning to become pregnant during the course of the study, or were unable to understand the study materials or interventions.

The study protocol was reviewed and approved by the Health Research Authority (HRA) NHS South Central Oxford B Research Ethics Committee (Reference 19‐SC‐0210), and prospectively registered on the ISRCTN registry (Reference ISRCTN14859844). The full protocol is available on https://www.phc.ox.ac.uk/research/participate/online-weight-loss-study-1. All participants provided consent to participate before they were enrolled on the trial.

### 2.2 Power calculation

The aim of the study was to compare each of the active interventions with the control (no intervention). We determined that after 8 weeks, a difference of 2 kg would represent a clinically meaningful effect. An a priori power calculation was conducted using SD of 3.9 kg^4^, ^5^ and assuming 90% power with two‐sided 5% level of significance and inflated to account for 20% drop out and adjusted for multiple comparisons using Bonferroni correction. This calculation determined that a minimum sample size of 528 (132 in each group) was required.

### 2.3 Participants and setting

We recruited participants from eight primary care practices located in four clinical research networks across the UK (Thames Valley and South Midlands; Kent, Surrey and Sussex; Yorkshire and Humber; West of England). Primary care providers searched their electronic registers for eligible individuals (aged ≥18 years and BMI ≥30 kg/m^2^ in the past 12 months) and sent a letter of invitation to all eligible patients inviting them to consider taking part in this online weight loss study. The invitation letter directed patients to the study website for more information or asked them to contact the study research team via email if they had any questions. The study website hosted all information about taking part in the study, including the participant information sheet. Once participants had read the information, they were able to complete a screening questionnaire to assess their eligibility to take part and to give their consent online.

### 2.4 Randomisation, allocation concealment, and blinding

To ensure allocation concealment, an independent statistician produced a computer generated randomisation list with equal allocation to groups using block sizes of four. This list was uploaded to a secure server that automatically sent an email that revealed whether participants had been allocated to the control (no intervention), NHS weight loss programme, Rosemary Online or Slimming World. This process ensured full allocation concealment.

Owing to the type of intervention, it was not possible to blind participants to treatment allocation. Body weight was self‐reported at baseline and after 8 weeks and entered online by participants themselves. Participants who did not respond to two follow‐up reminder emails to record their weight were contacted by the research team. Twelve participants asked for the follow‐up questionnaire to be completed over the phone with the researcher entering their data. The trial statistician was blinded to the group allocation until after the final analysis.

### 2.5 Interventions

#### 2.5.1 Control group

Participants randomised to the control group received no further contact until the final weight measurement was requested. They were not offered to access any of the programmes after their participation in the trial.

#### 2.5.2 Active intervention groups

Participants were randomised to one of the three active intervention groups, Rosemary Online, Slimming World Online and NHS Weight Loss Plan. Access to the first two was via an access code sent by email which, once activated, allowed them free access to an online weight loss programme accessible via a website or app. These programmes normally have a subscription charge but were provided free of charge to the participants. In the case of the NHS weight loss plan, which is freely available, participants were sent the website link. Participants randomised to Slimming World group had access to an online support team via a chat function on their website (Table S1). Participants rrandomised to Rosemary Online group had access to an online coach via a chat function and the standard protocols for these programmes include participants being proactively contacted at least once by the online support team. Access to the programmes was provided for 8 weeks for Rosemary Online, three calendar months for Slimming World (as this was a standard period for a trial of their programme and a change was not possible) and without limit for the NHS Weight Loss Plan as this is freely available.

### 2.6 Procedures

At baseline participants self‐reported their age, gender, ethnicity, height, and weight using an online form. In addition, to assess whether people were motivated to take part and ensure that participants could use a computer, they were asked to complete a baseline questionnaire (morningness/eveningness questionnaire).^6^, ^7^ Only those who completed the questionnaire were randomised.

An automated email and/or SMS was sent to randomised participants at 8 weeks, which included a link to an online form, in which participants self‐reported their current weight and completed an end of the study questionnaire about weight‐control practices over the preceding 8 weeks. Participants who did not respond to the request via email were contacted via telephone by a member of the research team who re‐sent the link to the questionnaire for the participant to complete or collected the follow‐up data verbally over the phone.

### 2.7 Outcomes

The primary outcome was change in self‐reported weight from baseline to 8 weeks. The secondary outcome was the proportion of participants losing ≥5% and ≥ 10% of their baseline weight.

### 2.8 Process measures

As a process measure, we aimed to examine the extent of engagement with programmes using data collected by the provider. Information on engagement was not available for the NHS Weight Loss Plan. For the other interventions, we collected data that was routinely available. This included the number of participants who did not activate vouchers for the programme and specifically, the number of times the website was accessed (Rosemary Online) and the number of times body weight was recorded on the website (Slimming World). We used data from the participant end of the study questionnaire to examine the proportion of people who reported continuing their weight loss attempt and their weight‐control practices at 8 weeks.

### 2.9 Statistical analysis

We followed a statistical analysis plan approved by the trial management group before database lock. The study was not designed to test for superiority between active interventions and they were not compared with each other. An analysis of covariance (ANCOVA) model with fixed effects for treatment arm and baseline weight was used to analyse the primary outcome using STATA 16.1. Analysis followed an intention to treat principle and in the primary analysis missing outcomes were imputed using Baseline Observation Carried Forward (BOCF). Additional sensitivity analyses were carried out to assess the robustness of the results to different missing data assumptions. Any baseline variable shown to be predictive of missing weight at 8 weeks was included as a fixed effect in a completer only analysis to estimate treatment effects that are valid under the missing at random (MAR) assumption. The proportion of patients losing at least 5% of baseline body weight was analysed using a log‐binomial regression model with the same adjustments as for the primary outcome. Intervention effects are presented for each weight loss programme, alongside a 95% confidence interval and a two‐sided *p*‐value. As the number of participants losing at least 10% body weight was too small descriptive statistics were used.

A sensitivity analysis was carried out on the primary outcome to assess the robustness of the results to different missing data assumptions and to assess the effect of excluding outliers. Additional sub‐group analyses were undertaken to assess whether the intervention effect differs by age and gender. These models were similar to those used for the primary outcome but with an additional interaction term between treatment arm and the moderator of interest (age or gender).

Associations between measures of engagement and weight loss at 8 weeks were analysed using the Spearman correlation coefficient.

## 3 RESULTS

**FIGURE 1:**
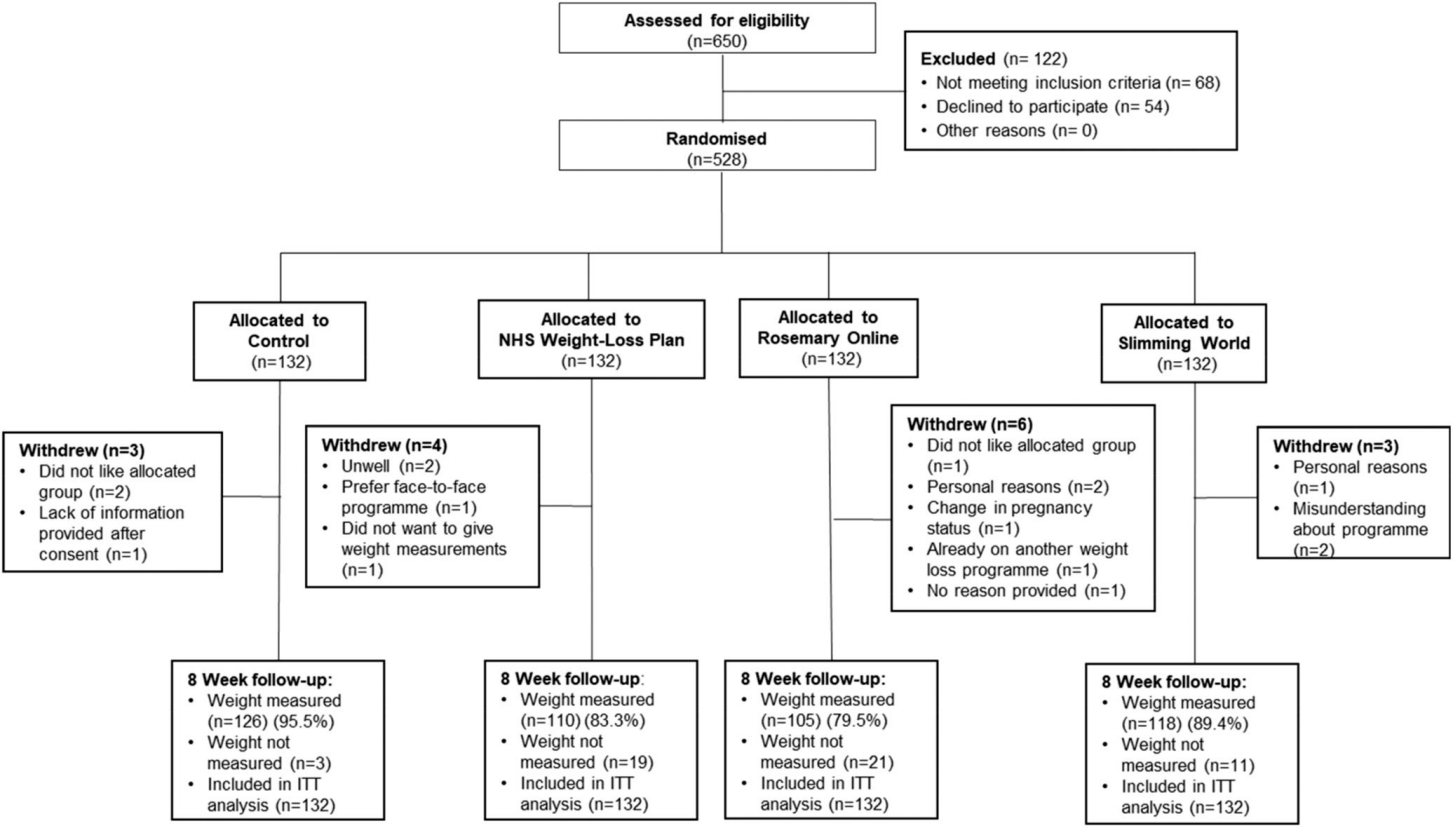
CONSORT 2010 flow diagram

Participants were recruited between 10 October 2019 and 5 December 2019. In total, 8235 letters were sent out to patients of eight GP practices. Of the 650 people who completed the screening questionnaire, 528 were eligible and gave consent to take part in the study, representing 6.4% of the potentially eligible population. Of the 528 participants enrolled, 16 participants withdrew from the trial. Follow‐up was completed on 4 March 2020 with data collected from 459 (86.9%) of participants who were randomised (Figure 1).

The average age of participants was 51 years (SD 15), 63.1% were female, 92.3% were white and 75.5% lived in an area of the 50% most deprived areas in England (Table 1).

**TABLE 1:** Baseline characteristics of participants enrolled on the online weight loss (OWL) study

	Control *n* 132	NHS Weight Loss Plan *n* 132	Rosemary Online *n* 132	Slimming World *n* 132	Overall *n* 528
Age (years)	49.6 (15.2)	52.7 (16.4)	49.2 (14.8)	52.6 (13.5)	51.0 (15.0)
Gender *n* (%)					
Male	44 (33.3%)	50 (37.9%)	49 (37.1%)	50 (37.9%)	193 (36.6%)
Female	88 (66.7%)	82 (62.1%)	82 (62.1%)	81 (61.4%)	333 (63.1%)
Other	0 (0.0%)	0 (0.0%)	1 (0.8%)	1 (0.8%)	2 (0.4%)
BMI (kg/m^2^)					
Mean (SD)	35.3 (4.5)	36.1 (5.3)	35.7 (5.0)	36.0 (5.5)	35.8 (5.1)
Median (IQR)	33.9 (31.7–37.9)	34.1 (32.0–38.8)	34.4 (32.0–38.2)	34.3 (32.1–38.0)	34.2 (32.0–38.5)
IMD^a^	*n* 118	*n* 113	*n* 111	*n* 111	*n* 453
% Above national median (*n*)	21.2 (25)	31.9 (36)	30.6 (34)	14.4 (16)	24.5 (111)
(%) Below national median (*n*)	78.8 (93)	68.1 (77)	69.4 (77)	85.6 (95)	75.5 (342)
Ethnicity *n* (%)					
White British	111 (84.1%)	116 (87.9%)	108 (81.8%)	112 (84.8%)	447 (84.7%)
Not white British	21 (15.9%)	16 (12.1%)	24 (18.2%)	20 (15.2%)	81 (15.3%)

^a^ Index of Multiple Deprivation (IMD) score was computed for participants who provided postcode. IMD ranks geographical areas in the UK on seven indices: income, employment, health deprivation and disability, education, crime, barriers to housing and services, and living environment. These ranks are grouped into deciles which were used for analysis with lowest decile representing the most deprived areas and highest decile representing the least deprived areas.^23^

### 3.1 Prim**ary Outcome**

On average, all groups lost weight over the course of the study. Mean (95% confidence Interval) weight change after 8 weeks between control group and NHS Weight Loss Plan was −0.4 (−1.3 to 0.5), between control group and Rosemary Online was −1.5 (−2.3 to −0.6) and control group and Slimming World was −0.8 (−1.7 to 0.1). Only the Rosemary Online group showed a statistically significantly greater weight loss compared with the control group. Sensitivity analyses using only those who provided follow‐up data did not alter the conclusion, nor did the exclusion of outliers, defined as weight change more than three standard deviations away from the mean (Table 2). A sub‐group analysis showed that the intervention effect did not differ by age or gender (Table S2).

**TABLE 2:** Weight loss outcomes

	Control^a^	NHS Weight Loss Plan	Rosemary Online	Slimming World
Primary outcome (BOCF)	*n* 132	*n* 132	*n* 132	*n* 132
Weight change (kg)^b^	−0.8 (3.0)	−1.3 (3.9)	−2.3 (3.8)	−1.7 (3.4)
Adjusted difference (95% CI)^c^		−0.4 (−1.3 to 0.5)	−1.5 (−2.3 to −0.6)	−0.8 (−1.7 to 0.1)
*p*‐value^d^		0.364	0.001	0.070
Sensitivity analyses				
Completers only	*n* 126	*n* 110	*n* 105	*n* 118
Weight change (kg) (SD)^b^	−0.9 (3.1)	−1.5 (4.3)	−2.9 (4.0)	−1.9 (3.5)
Adjusted difference (95% CI)^e^		−0.6 (−1.6 to 0.4)	−2.0 (−3.0 to −1.1)	−1.0 (−1.9 to −0.0)
*p* value^c^		0.217	<0.001	0.045
Excluding outliers^f^	*n* 130	*n* 127	*n* 128	*n* 131
Weight change (kg)^b^	−0.6 (2.3)	−1.1 (2.6)	−1.9 (3.1)	−1.5 (3.0)
Adjusted difference (95% CI)^g^		−0.5 (−1.2 to 0.1)	−1.4 (−2.0 to −0.7)	−0.9 (−1.6 to −0.2)
*p*‐value^d^		0.117	<0.001	0.007

^a^ Reference group.

^b^ Mean and standard deviation.

^c^ Estimated from an analysis of covariance model adjusted for baseline weight.

^d^ Compared with control group.

^e^ Estimated from an analysis of covariance model adjusted for baseline weight and age at screening.

^f^ Outliers were defined as individuals with weight change more than three standard deviations away from the mean.

^g^ Estimated from an analysis of covariance model adjusted for baseline weight.

### 3.2 Secondary outcomes

**FIGURE 2:**
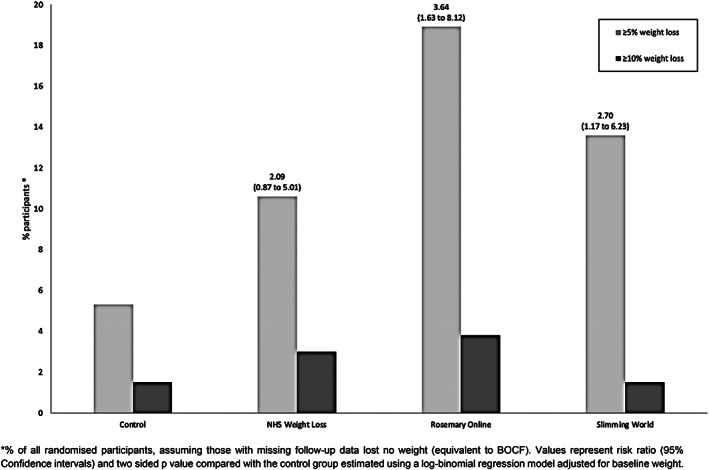
Proportion of participants reporting >5 and > 10% weight loss. Values represent risk ratio (95% confidence intervals) and two sided *p*‐value compared with the control group estimated using a log‐binomial regression model adjusted for baseline weight

For the purposes of this analysis, It was assumed participants who were not followed‐up lost no weight (BOCF). The proportion of participants who lost at least 5% of baseline body weight was significantly higher in both the Rosemary Online (RR 3.64 [95% CI 1.63–8.12], *p* 0.002) and Slimming World (RR 2.70 [95% CI: 1.17–6.23], *p* 0.020) groups compared with the control group. There was no evidence that the proportion of participants in the NHS Weight Loss Plan group who lost at least 5% baseline weight was different from the control group (RR 2.09 [95% CI: 0.87–5.01] *p* 0.098). Only five or fewer participants in each group lost ≥10% (Figure 2).

### 3.3 Engagement

Eighty‐two percent of randomised participants in the Slimming World group, and 89% in the Rosemary Online group activated the vouchers that provided access to the programmes free of charge. No voucher was needed to access the NHS Weight Loss Plan; therefore, we have no objective measure of the proportion of participants allocated to this group who initially engaged with the programme. Among participants who completed the end of the study questionnaire (*n* = 459, 86.9%) 22.0% reported not starting their allocated programme (Table 3).

**TABLE 3:** Self‐reported participant adherence and experience

	Control group	NHS Weight Loss Plan	Rosemary Online	Slimming World
Number of responses	*n* 101	*n* 55	*n* 55	*n* 46
Reasons for not following a programme	% of total responses (*n*)	% of total responses (*n*)	% of total responses (*n*)	% of total responses (*n*)
At the beginning of the study I was asked to continue my usual routine	89.1 (90)	9.1 (5)	1.8 (1)	2.2 (1)
I did not have time or resources to follow the programme	1.0 (1)	27.3 (15)	29.1 (16)	17.4 (8)
I did not start the allocated programme	1.0 (1)	18.2 (10)	21.8 (12)	26.1 (12)
I started the programme and later stopped before reaching my goal	0.0 (0)	21.8 (12)	14.5 (8)	28.3 (13)
Personal reasons (e.g. sickness, holidays, mental load)	2.0 (2)	12.7 (7)	10.9 (6)	6.5 (3)
Did not like the allocated programme	0.0	0.0	9.1 (5)	8.7 (4)
Because it was over the Christmas period	1.0 (1)	3.6 (2)	1.8 (1)	4.3 (2)
Other reasons	5.9 (6)	7.3 (4)	10.9 (6)	6.5 (3)

Participants in the Rosemary Online group logged onto the website on a mean of 12.8 occasions (SD 25.0, median 6.0, range 1–150). Participants in the Slimming World Online group reported their weight on the website on a mean of 4.8 occasions (SD 3.0, median 5.0, range 0–9). There was no association between these indirect measures of engagement with the programmes and weight change at 8 weeks for either Rosemary Online (**ρ** = −0.186, *p* = 0.078) or Slimming World (**ρ** = −0.157, *p* = 0.118) programmes (Figure S1).

### 3.4 Reasons for not following a weight loss programme and perceived effectiveness

Among participants who completed the end of the study questionnaire the proportion of participants who reported following a structured weight loss programme at 8 weeks was two to three times greater in the active intervention groups than control (19.8%); NHS Weight Loss Plan (49.1%), Rosemary Online (46.2%), and Slimming World (60.2%). The most common reasons reported for not following a weight loss programme were not having the time or resources. Other explanations included personal reasons, not liking the allocated programme and the study period including the Christmas holidays for some participants (Table 3).

## 4 DISCUSSION

All groups lost small amounts of weight, but only the Rosemary Online programme group lost significantly more weight than the control group. Participants who were randomised to the Rosemary Online group lost 1.5 kg more than the control group, who lost 0.8 kg, and were over three times more likely to have lost at least 5% of their body weight during the initial 8 weeks compared with the control (no intervention group). There was no evidence that weight loss in the Slimming World group was statistically different than the control group (mean difference − 0.8 kg), but participants were almost three times more likely to have lost at least 5% of their body weight during the 8 weeks than the control group. Weight change in the NHS Weight Loss Plan was not significantly different from control.

### 4.1 Strengths and limitations

This study recruited a general population of people living with obesity in four different geographical regions of England with a good mix of socioeconomic status. We only excluded people that would usually not be recommended to follow a weight loss programme or people who did not have access to online services. More than a third of participants were men, which is much higher than observed in GP referrals to face to face weight loss groups in the UK, where only one in 20 attendees are men and somewhat higher than in other randomised controlled weight loss trials with an average of 27% of male participants.^8^ It is difficult to study the impact of largely unsupported weight loss attempts since the procedures associated with participation in a trial can act as an intervention. However, we conducted the trial remotely with no personal contact with the research team to minimise these effects.

The main limitation of this study is that the outcome measures were not objectively measured. There is a risk that participants did not accurately report their current weight, either at baseline or at follow‐up, even though they were given specific instructions about when and how to weigh themselves. However, since this was a randomised controlled trial any bias should be equally distributed across the groups. Although the overall loss to follow up (13.0%) was much lower than in many weight loss studies there were some differences between groups. Unusually, the attrition was greater among intervention compared to control groups, but the sensitivity analysis of completers did not change the interpretation of the primary outcome. The follow‐up period was short but there is evidence that early weight loss is a good predictor of longer‐term outcomes in behavioural weight loss interventions^9^, ^10^ and given the small weight losses observed, a longer trial which would require a larger sample size due to increased variability in weight change, is hard to justify.

It was not possible to blind study participants to their treatment allocation, but trial statisticians were blinded until the final analysis was complete. Furthermore, given that this study took part entirely online, with outcome measures self‐reported this could help to reduce observer bias. In studies of interventions that are widely available it is common and unavoidable that contamination may occur in the treatment allocation, and in this pragmatic trial we did not seek to discourage help‐seeking behaviour. We had limited measures of engagement with the programmes which may explain the lack of association between our markers of engagement and weight loss, when most weight loss studies show a strong positive association between engagement with the programme and weight loss.^11^, ^12^, ^13^, ^14^ The challenge of using routinely collected data as a process measure highlights the importance of standard process measures when comparing different programmes in real life.

### 4.2 Comparison with other studies

Several recent systematic reviews have reported that digital weight loss programmes are more effective than usual care or minimal intervention.^15^, ^16^, ^17^, ^18^, ^19^ This study tested largely unsupported digital programmes in a controlled trial. Innes et al. previously randomised 25 people to follow the NHS Weight Loss Plan for 12 weeks in combination with a free gym membership.^20^ Mean weight loss in this group (−4.2 kg) was greater than we observed in the present study, and was significantly greater than the control group (−1.17 kg), who received a free gym membership alone.^20^ The reasons for the greater weight loss are unknown, but likely relate to differences in the way participants were recruited to take part between the studies. Innes et al. recruited participants who had proactively responded to an advertisement to take part, reflecting some intrinsic motivation for weight loss. However, in our study, we excluded people who were already following a weight loss programme, and hence the sample reflects participants who have simply been prompted to consider weight loss on receipt of a letter from their doctor. While receiving this letter of invitation may create momentary motivation to lose weight, this trial suggests it may be insufficient to encourage high levels of engagement and adherence with the programme. The fact that participants were randomly allocated to one of the programmes and were not involved in a conversation about the treatment programme might also have impacted on their motivation.

Overall, the weight loss in each of the intervention groups was modest and less than reported for many other digital interventions. A review of effective digital interventions found that personal support in the form of communication and feedback from a counsellor was an important component of successful digital weight loss interventions.^3^ Here, the NHS Weight Loss Plan offered no in‐person support, Slimming World Online had the opportunity for online chat and Rosemary Conley Online only had one proactive contact, although there were opportunities to seek support online.

### 4.3 Implications for practice

It is clear from this and other analyses that not all weight loss programmes are effective and the pace of change in the digital market is high with new programmes being developed and others being updated. All three programmes tested in this study have had significant updates or replacements since the study ended. The NHS Weight Loss Plan has had the content enhanced and updated from a stand‐alone web‐based programme to include app provision. The Rosemary Online programme tested in this study programme is no longer available. Some of the components used in the Rosemary Online programme tested here are available as GetSlim. The content and support of the Slimming World programme has been enhanced as part of ongoing programme development.

It may not be possible to test every intervention before roll‐out, but programmes need to be strongly grounded in evidence of effective components and ongoing monitoring is essential so that services with limited effectiveness can be enhanced or ceased.

The low uptake of the interventions in this trial should prompt caution when using largely unsupported online/digital weight management programmes in routine primary care. Response to the GP letters was low and while the additional processes of the trial cannot be ignored as a possible deterrent, we took steps to ensure the burden was minimal. Previous trials suggest that uptake is much higher when a referral is offered in‐person during a consultation with a healthcare professional. In a trial of brief opportunistic interventions in consultations with GPs 77% of patients accepted the offer of a referral and of these 40% of patients attended the programme,^21^ compared with around 10% uptake when invitations are by letter only.^22^

## 5 CONCLUSION

In conclusion, this study shows that referral to these online weight loss programmes with little or no in‐person support during the programme is, at best, only marginally superior to no intervention.

## CONFLICT OF INTEREST

Rosemary Online and Slimming World granted free access to their programmes for participants randomised to the respective study arm. Michaela Noreik, Claire D. Madigan, Nerys M. Astbury, Rhiannon M. Edwards, Ushma Galal, Jill Mollison, Fitsum Ghebretinsea declare no other conflict of interest. Susan A Jebb received payment to the institution from Oviva Ltd. for a presentation unrelated to this project.

## AUTHOR CONTRIBUTION

Claire D. Madigan, Nerys M. Astbury, Susan A. Jebb developed the concept for the study and wrote the protocol. Claire D. Madigan and Michaela Noreik prepared the study documents and coordinated the HRA and ethics application. Ushma Galal, Jill Mollison, Fitsum Ghebretinsea were the trial statisticians. Michaela Noreik and Rhiannon M. Edwards ran the trial and collected study data. Michaela Noreik drafted the manuscript for publication, with input from Susan A. Jebb and Nerys M. Astbury. All authors revised the manuscript and approved the final manuscript.

## Supporting information


**Table S1** Features included in each of the active interventions
**Table S2** Sub‐group analysis by age and gender
**Figure S1** Association between engagement and weight loss at 8 weeks
