# Supplementary material for: Testing the short‐term effectiveness of primary care referral to online weight loss programmes: A randomised controlled trial
Source: Clin Obes. 2021 Oct 6;11(6):e12482. doi: 10.1111/cob.12482 (PMC9285966; doi:10.1111/cob.12482)
Supplement: Supplementary file 1 — Table S1 Features included in each of the active interventions Table S2 Sub‐group analysis by age and gender Figure S1 Association between engagement and weight loss at 8 weeks [file COB-11-0-s001.docx]

## Supplementary Table 1 Features included in each of the active interventions

| **Name** | **Features** |
| --- | --- |
| **NHS Weight Loss Plan** | - 12-week plan - Group help features - Easy meals app - Links to other items like couch to 5k |
| **Rosemary Online** | - Tracking tools e.g. weight monitoring, food and exercise diary - Online community - Online coaches - Recipes - Exercise ideas/videos - Mobile friendly (includes App) - Daily motivational review |
| **Slimming World** | - Tracking and planning tools e.g. weight monitoring, food and activity - Self-led behaviour change tools - Online community - Live chat support - Recipes - Motivational content - Mobile friendly (includes App) |

**Supplementary Table 2** Sub-group analysis by age and gender

|  |  | **Control¹** | **NHS Weight Loss Plan** | **Rosemary Online** | **Slimming World** |
| --- | --- | --- | --- | --- | --- |
|  |  | **n 132** | **n 132** | **n 132** | **n 132** |
| **Age** |  |  | | | |
| **Treatment effect (95% CI]²** | **n** |  |  |  |  |
| 18-43 years | 171 |  | -0.2 (-1.7 to 1.3) | -1.5 (-2.9 to -0.1) | 0.4 (-1.2 to 2.0) |
| 44-58 years | 181 |  | -0.3 (-1.8 to 1.3) | -1.1 (-2.6 to 0.3) | -2.0 (-3.3 to -0.6) |
| >58 years | 176 |  | -0.6 (-2.1 to 0.8) | -1.8 (-3.4 to -0.2) | -0.5 (-2.0 to 1.1) |
| **P value²** |  | 0.161 | | | |
| **Gender^3^** |  |  | | | |
| **Treatment effect (95% CI)³** | **n** |  |  |  |  |
| Male | 193 |  | -0.3 (-1.7 to 1.2) | -1.7 (-3.1 to -0.3) | -0.5 (-2.0 to 0.9) |
| Female | 333 |  | -0.5 (-1.5 to 0.6) | -1.2 (-2.3 to -0.2) | -1.0 (-2.1 to 0.1) |
| **P value^4^** |  | 0.750 | | | |

¹ Reference group
² For the interaction term in analysis of covariance model including randomised group, baseline weight, age and age x randomised group interaction
³ Participants who reported other gender were excluded from the subgroup analysis.

^4^ For the interaction term in analysis of covariance model including randomised group, baseline weight, gender, and gender x randomised group interaction

**Supplementary Figure 1** Association between engagement and weight loss at 8 weeks

n = 91, Spearman's correlation coefficient = -0.186, p = 0.078 n = 100, Spearman's correlation coefficient = -0.157, p = 0.118
